# The Association Between Humidex and Daily Outpatient Visits for Pediatric Respiratory Diseases in Shijiazhuang, China: A Time Series Analysis

**DOI:** 10.3389/ijph.2025.1607752

**Published:** 2025-03-17

**Authors:** Xixi Du, Fengge Chen, Mingyang Guan, Feng Li, Hui Kang, Yang Wang

**Affiliations:** ^1^ Department of Public Health Monitoring and Evaluation, Shijiazhuang Center for Disease Control and Prevention, Shijiazhuang, Hebei, China; ^2^ School of Public Health, North China University of Science and Technology, Tangshan, Hebei, China; ^3^ Research Base for Environment and Health in Shijiazhuang, Chinese Center for Disease Control and Prevention, Shijiazhuang, Hebei, China; ^4^ Shijiazhuang Municipal Health Commission, Shijiazhuang, Hebei, China

**Keywords:** humidex, respiratory disease, child, time series analysis, DLNM

## Abstract

**Objectives:**

At present, most studies have focused on the effects of temperature or humidity on children’s health, while relatively few have explored the combined effects of temperature and humidity on children’s health. We aimed to examine the impact of humidex, a comprehensive temperature and humidity index, on the outpatient department of respiratory diseases in children.

**Methods:**

Daily outpatient visits for pediatric respiratory disorders, meteorological conditions, and air pollution in Shijiazhuang were recorded. From 2014 to 2022, we evaluated the impact of humidex on outpatient visits for respiratory disorders in children using a distributed lag non-linear model (DLNM). The model controlled air pollution (PM_2.5_, NO_2_, and SO_2_) and wind velocity, as well as day of week, seasonality, and long-term trend. In addition, stratified analysis was performed according to different genders, ages, and disease types.

**Results:**

Humidex and the outpatient exposure-response curve of children’s respiratory diseases showed a “V” type. The cumulative relative risks (CRR) of extremely high and low humidex were 1.124 (95% confidence interval [CI] = 1.030–1.228) and 1.344 (95% CI = 1.136–1.590), respectively. The burden of respiratory diseases in children attributed to non-optimal humidex was 13.96% (95% empirical CI[eCI] = 7.81–19.33%), most of which was attributed to low humidex, with an AF of 12.54% (95% eCI = 5.94–18.32%), and only 1.42% (95% eCI = 0.19–2.48%) was due to high humidex.

**Conclusion:**

Low humidex exposure significantly increased the risk of respiratory illnesses in children, and children aged 7–14 were more susceptible to low humidex.

## Introduction

Climate change refers to long-term shifts in temperatures and weather patterns. Natural causes may cause these changes, but since the 19th century, human activities have been the main driver of climate change characterized by global warming [1]. Respiratory system disease is a common and frequent disease. Its pathogenic factors are complex and diverse, and it is difficult to prevent and control, which seriously affects people’s health and life safety [2]. In 2019, respiratory diseases had become the third leading cause of death worldwide, accounting for 4 million global deaths [3]. Studies indicated that the atmospheric environment, including external meteorological changes, directly impacted respiratory diseases, especially temperature [4–6]. A study based on 272 major cities in China showed that 10.57% of respiratory deaths can be attributed to non-optimal (cold and hot) temperatures [7]. Therefore, to effectively prevent respiratory diseases and reduce the burden of diseases caused by them, it is essential to clarify the impact of environmental risk factors such as temperature on respiratory diseases. In addition, there are significant differences in the susceptibility of different populations to temperature [8]. Children seem more susceptible to temperature changes than adults due to the continuous development of their respiratory and immune systems [4, 9].

Although many studies have explored the relationship between ambient temperature and respiratory diseases in children, the results of existing studies are inconsistent [10–13]. This may be due to the heterogeneity of studies in different regions and the different confounding factors (such as air pollutants and meteorological factors) included in the study, resulting in a large difference in the results. In addition, humidity is also closely related to the occurrence and development of respiratory diseases [14]. Low humidity is positively correlated with the incidence of respiratory diseases in children [11]. The bulk of current studies examine just the single effects of temperature or humidity after correcting for confounding variables. However, there may be potential interactions between temperature and humidity [15–17], and the combined impacts of meteorological elements may alter the effects of a single factor. Furthermore, due to the influence of topography, altitude, and climate, the humidity in different regions is quite different [18], and humidity significantly impacts the temperature perceived by humans. Even if the ambient temperature is similar and the humidity is different, it will lead to different somatosensory temperatures [19]. Therefore, using only ambient temperature as a representative indicator to predict the impact of weather on human health may ignore the role of humidity. We urgently need a comprehensive indicator representing temperature and humidity to indicate the health risks caused by weather changes.

Humidex was initially proposed by the Canadian Meteorological Agency. It integrates the impacts of temperature and humidity, allowing for a more accurate evaluation of the relationship between temperature, humidity, and human health [20]. Humidex was used to evaluate the impact of temperature and humidity on childhood illnesses [14, 21, 22].

Based on the aforementioned concerns, we conducted this study with three primary goals: First, we will investigate the effect of humidex on outpatient visits for respiratory disorders in children. Second, we will identify vulnerable populations and sensitive diseases through stratified analysis. Furthermore, we want to know whether the comprehensive measure, humidex, is more suitable as a predictor than the daily average temperature.

## Methods

### Study Area

Shijiazhuang is the capital of Hebei Province, located in North China (38°04′N, 114°29′E). Shijiazhuang has a typical temperate continental monsoon climate with four distinct seasons, with most rain falling in the summer and autumn. During the investigation, temperatures in Shijiazhuang ranged from −10.2°C–34.7°C. This study’s outpatient, meteorological, and air pollution data come from the same place.

### Data Collection

The study acquired daily outpatient visit data from Hebei Children’s Hospital between 1 January 2014, and 31 December 2022. Outpatient data are short-term effect outcome indicators. Compared with hospitalization and death indicators, it is more sensitive to reflecting the acute impact of environmental risk factors on respiratory diseases and timely feedback on health information [23]. Hebei Children’s Hospital is a tertiary general hospital with a central location and easy access to transportation. It serves as the region’s medical service center and the largest hospital for pediatric admissions in Shijiazhuang. In particular, the Department of Respiratory Medicine, as the provincial medical key discipline of the hospital, is highly skilled in treating children’s respiratory diseases. The annual outpatient volume for respiratory disorders surpasses 200,000 people, and the daily outpatient reception capacity has yet to approach saturation. As a result, the hospital is well represented and can properly reflect the incidence of common respiratory disorders in children in Shijiazhuang.

We excluded patients with non-Shijiazhuang household registration, age over 14 years old, and non-Shijiazhuang urban resident addresses when collecting data. Outpatient visit causes were classified using the International Statistical Classification of Diseases, 10th revision (ICD-10), which included respiratory disease causes (RESP, codes J00-J99 and R04-R9.3), acute upper respiratory infections (AURIs, codes J00-J06), influenza and pneumonia (FLU&PN, codes J09-J18), and acute lower respiratory infections (ALRIs, codes J20-J22). We divided outpatient visits by gender and age group (0–6 and 7–14 years).

Meteorological data were obtained from the Shijiazhuang Meteorological Bureau, including daily average temperature, daily average relative humidity, and mean wind velocity. The Shijiazhuang Environmental Monitoring Center provided daily data on air pollution, including 24-hour mean fine particulate matter (PM_2.5_), particulate matter with particle size below 10 microns (PM_10_), nitrogen dioxide (NO_2_), sulfur dioxide (SO_2_), and maximum eight-hour mean concentration of ozone (O_3_) concentrations.

Humidex, proposed by Canadian meteorologists Richardson and Masterton, is an index that combines the effects of temperature and humidity; it can better characterize the actual temperature that humans feel [20]. Humidex is dimensionless but can be expressed as a drying temperature of °C. For example, when the temperature is 30°C, and the calculated humidex is 40, indicating that the feeling at this time is similar to the drying temperature of 40°C. Meanwhile, humidex increases with temperature and humidity. The daily humidex calculation method in Shijiazhuang is based on the strategy offered by CSGNetwork (http://www.csgnetwork.com/) in California, which is as follows (Formula 1):

$$Humidex=T+\frac{5}{9}\left[6.11\times 10^{\left(\frac{7.5\times T}{237}+T\right)}\right]\times \frac{RH}{100}-10$$ (1)

Where *T* is the daily average temperature (°C); *RH* is the daily average relative humidity (%).

### Data Quality Control

The integrity, validity, and accuracy of the data were all constrained by quality control. We identified and deleted duplicate data on key factors, such as date of birth, permanent address, date of visit, and reason of visit. We also checked whether the ICD code was correct according to the reason for the visit and corrected it to ensure the correct diagnosis classification. The meteorological data came from a stable and reliable monitoring system without omission. The overall percentage of missing data for air pollutants was less than 1.5%, and missing values were interpolated using the average of three adjacent values.

### COVID-19 Lockdown Classification

Data were collected from 1 January 2014 to 31 December 2022. Given the impact of the COVID-19 epidemic in 2020–2022, which may raise or reduce daily hospital outpatient volume, it is treated as a confounding factor. According to the epidemic risk level and control measures of Shijiazhuang, we have determined six lockdown periods: 1 January 2020–31 March 2020, 3 January 2021–21 February 2021, 23 October 2021–13 November 2021, 25 August 2022–10 September 2022, 13 September 2022–5 October 2022, and 27 October 2022–1 December 2022.

### Statistical Analysis

We used a distributed lag nonlinear model (DLNM) to investigate the relationship between humidex and cause-specific outpatient visits for respiratory illnesses in children. Meanwhile, spearman’s correlation coefficient was used to examine the relationship between humidex and meteorological parameters, as well as between humidex and air pollutants. To avoid multicollinearity, we included components with correlation analysis results in *r*_s_ ≤0.7 in the model [24]. We used natural cubic regression smoothing to account for seasonality, long-term trends, meteorological factors, and air pollution. Our calculations minimized the Akaike’s Information Criterion (AIC) [25] value of DLNM by allowing 8 degrees of freedom (df) per year for long-term trends, 3 df for meteorological parameters, and 3 df for air pollution. Finally, we obtained the fundamental model shown as follows (Formula 2):

$$\mathrm{Log}\left(\mu t\right)=\beta {\text{Humidex}}_{t,l}+\text{ns}\left(\text{time},8\right)+\text{ns}\left(\text{wind},3\right)+\text{ns}\left(\text{pollutants},3\right)+\text{DOW}+\text{nCov}+\alpha$$ (2)

Where *t* is the day of observation; *μt* is the daily number of children with respiratory diseases on day t; *β* is the matrix coefficient; *Humidex*_*t,l*_ is the cross basis in DLNM; *ns* is the natural cubic spline function in R; *time* represents the long-term trend of time; *wind* represents mean wind velocity; *pollutants* represent different air pollutants, including, PM_2.5_, NO_2_, SO_2_; *DOW* is a dummy variable for weeks; *nCov* is a binary variable, indicating the impact of COVID-19; α is the intercept of the model. In addition, we set the longest lag days to 21 days.

In this study, the reference value of humidex is related to the minimum risk of childhood respiratory illnesses (MRH). With MRH (value is 27) as the cut-off value, we divided humidex into two levels: low and high. We defined the 5th (value is −2) and 95th (value is 38) percentiles of humidex as extremely low and high humidex and utilized relative risk (RR) and 95% confidence interval (CI) to evaluate the influence of humidex on respiratory diseases in children. Furthermore, we conducted stratified analysis according to gender, age, and disease type, and used the Z test (Supplementary Formula S1) to compare differences between groups.

To prove whether humidex or temperature was a better indication of public health intervention, we calculated the daily average temperature using the same criteria as humidex. We additionally accounted for the influence of daily average relative humidity in the model, and the model formula was as follows (Formula 3):

$$\mathrm{Log}\left(\mu t\right)=\beta {\text{Temp}}_{t,l}+\text{RH}+\text{ns}\left(\text{time},8\right)+\text{ns}\left(\text{wind},3\right)+\text{ns}\left(\text{pollutants},3\right)+\text{DOW}+\text{nCov}+\alpha +\text{Temp}\times \text{RH}$$ (3)

Where *Temp* is the daily average temperature; *RH* is the average daily relative humidity; *Temp* × *RH* is the interaction term of daily average temperature and daily average relative humidity; others are the same as Formula 2.

Then, using a backward perspective within the DLNM framework, we estimated the attributable fraction (AF) and attributable number (AN) of outpatient visits for respiratory diseases in children exposed to humidex, taking into account risk at dayt as the prior period’s (t-l_0_, ……, t-L) cumulative exposure effects. Monte-Carlo simulations were used to calculate the 95% empirical confidence intervals (eCI) of the attributable fraction and number, assuming a multivariate normal distribution of the best linear unbiased predictions of the deduced coefficients [26, 27]. The humidex was further divided into low and high humidex by using MRH. In addition, we analyzed the attribution burden in subgroups divided by age, gender, and disease type using the above model. The calculation formulas were as follows (Formulas 4 and 5):

$${AF}_{x,t}=1-\exp\left(-\sum_{{l=l}_{0}}^{L}\beta_{x_{t-l},l}\right)$$ (4)

$${AN}_{x,t}={AF}_{x,t}\times n_{t}$$ (5)

Where ${AF}_{x,t}$ and ${AN}_{x,t}$ are the attributable fraction and number of cases at day_t_, respectively; *β*_x_ is the relative risks at day_t_; *L* is the maximum lag days; *l*_*0*_ is the minimum lag days; $n_{t}$ is the number of children with respiratory diseases on day_t_.

### Sensitivity Analysis

To assess the robustness of our model, sensitivity tests were performed: 1) the df of time was modified to df = 8–9; 2) the df of wind was changed to df = 3–5. Relevant results can be found in Supplementary Material (Supplementary Table S1). Statistical studies were conducted using R Statistical Software (version 4.3.2; The Free Software Foundation’s GNU Public License, Vienna, Austria). We performed two-tailed tests, and *P* < 0.05 was considered statistically significant.

## Results

### Descriptive Statistics

Table 1 describes the daily outpatient information on children’s respiratory diseases from 1 January 2014 to 31 December 2022. We collected 2,029,361 cases. Among them, 1,192,224 boys were treated for respiratory diseases, accounting for 58.7% of the total outpatients. Children aged 0–6 years had the largest number of visits for respiratory diseases, with 1,753,562 cases. In the subgroup of disease type, the highest group was AURIs with 732,793 cases, followed by ALRIs with 311,116 cases, and the smallest group was FLU&PN.

**TABLE 1:** Basic situation of outpatient volume of respiratory diseases in children (Shijiazhuang, China, 2014–2022).

Variables	N	Percent (%)
Daily outpatient visits		
RESP	2,029,361	100%
Child’s gender		
Boy	1,192,224	58.7%
Girl	837,137	41.3%
Age (in years)		
0–6	1,753,562	86.4%
7–14	275,799	13.6%
Disease types		
AURIs	732,793	36.1%
FLU&PN	284,486	14.0%
ALRIs	311,116	15.3%

Notes: N, number of outpatient children with respiratory diseases; RESP, respiratory diseases; AURIs, acute upper respiratory infections; ALRIs, acute lower respiratory infections; FLU&PN, influenza and pneumonia.

Table 2 presents the descriptive data of humidex, meteorological factors, and air pollutants. Humidex ranged from −10.2 to 45.0. The number of extremely low humidex (<−2) days was 163 days, 4.9% of the total number of days; the extremely high humidex (>38) was 136 days, 4.1% of the total days. Moreover, the daily average mean temperature, relative humidity, and wind speed were 15.8°C, 56.0%, and 1.8 m/s, respectively. During the study period, the daily average concentrations of PM_10_, O_3_, PM_2.5_, NO_2_, and SO_2_ were 109, 87, 54, 41, and 16 μg/m^3^, respectively.

**TABLE 2:** Description summary of humidex, meteorological factors, and air pollutants (Shijiazhuang, China, 2014–2022).

Variables	Mean ± SD	Min	P_25_	P_50_	P_75_	Max
Meteorological factors						
Humidex	17.32 ± 13.37	−10.2	5.0	16.7	29.0	45.0
Mean temperature (°C)	14.88 ± 10.69	−10.2	5.0	15.8	24.6	34.7
Relative humidity (%)	55.86 ± 20.25	7.0	40.0	56.0	72.0	100.0
Wind speed (m/s)	1.88 ± 0.79	0.3	1.3	1.8	2.2	6.5
Air pollutants (μg/m^3^)						
PM_10_	135.72 ± 100.59	13	70	109	167	867
PM_2.5_	76.52 ± 70.03	6	33	54	94	625
O_3_	96.64 ± 59.69	2	51	87	136	310
NO_2_	46.14 ± 24.66	7	28	41	59	188
SO_2_	28.02 ± 33.63	2	9	16	33	307

Notes: SD, standard deviation; Min, Minimum; P_25_, 25th percentile; P_50_, 50th percentile; P_75_, 75th percentile; Max, Maximum; PM_10_, particulate matter less than 10 μm; PM_2.5_, particulate matter less than 2.5 µm; O_3_, ozone; NO_2_, nitrogen dioxide; SO_2_, sulphur dioxide.

Supplementary Figure S1 presented the time-series distribution of outpatient visits for children’s respiratory diseases, humidex, temperature, and humidity from 2014 to 2022 in Shijiazhuang. The total number of daily outpatient visits for children’s respiratory diseases, humidex, and temperature had seasonal trends. Among them, the temperature exhibited the same pattern as the humidex, which was reduced in winter and more elevated during the summer.

To avoid co-linearity in the model, we used spearman’s approach to examine the correlation between humidex and meteorological indicators or air pollutants (Table 3). According to the results of correlation analysis, the possible confounding factors were identified, which were wind speed (*r*_s_ = 0.14, *P* < 0.01), PM_2.5_ (*r*_s_ = −0.36, *P* < 0.01), NO_2_ (*r*_s_ = −0.46, *P* < 0.01), SO_2_ (*r*_s_ = −0.34, *P* < 0.01). Furthermore, humidex was determined using the daily average temperature and daily average relative humidity. As a result, the regression model did not account for the daily average temperature or relative humidity levels.

**TABLE 3:** Spearman’s correlation coefficients of humidex, meteorological parameters, and air contaminants (Shijiazhuang, China, 2014–2022).

Variables	Humidex	Temperature	Humidity	Wind speed	PM_10_	PM_2.5_	O_3_	NO_2_	SO_2_
Humidex	1.00								
Temperature	0.99**	1.00							
Humidity	0.26**	0.16**	1.00						
Wind speed	0.14**	0.19**	−0.41**	1.00					
PM_10_	−0.35**	−0.33**	−0.03	−0.30**	1.00				
PM_2.5_	−0.36**	−0.37**	0.20**	−0.43**	0.92**	1.00			
O_3_	0.78**	0.81**	−0.04*	0.33**	−0.27**	−0.39**	1.00		
NO_2_	−0.46**	−0.44**	−0.12**	−0.34**	0.70**	0.65**	−0.42**	1.00	
SO_2_	−0.34**	−0.29**	−0.32**	−0.24**	0.67**	0.59**	−0.23**	0.70**	1.00

Notes: *P < 0.05; **P < 0.01; PM_10_, particulate matter less than 10 μm; PM_2.5_, particulate matter less than 2.5 µm; O_3_, ozone; NO_2_, nitrogen dioxide; SO_2_, sulphur dioxide.

### Relationship Between Humidex and Outpatient Visits for Pediatric Respiratory Diseases

**FIGURE 1:**
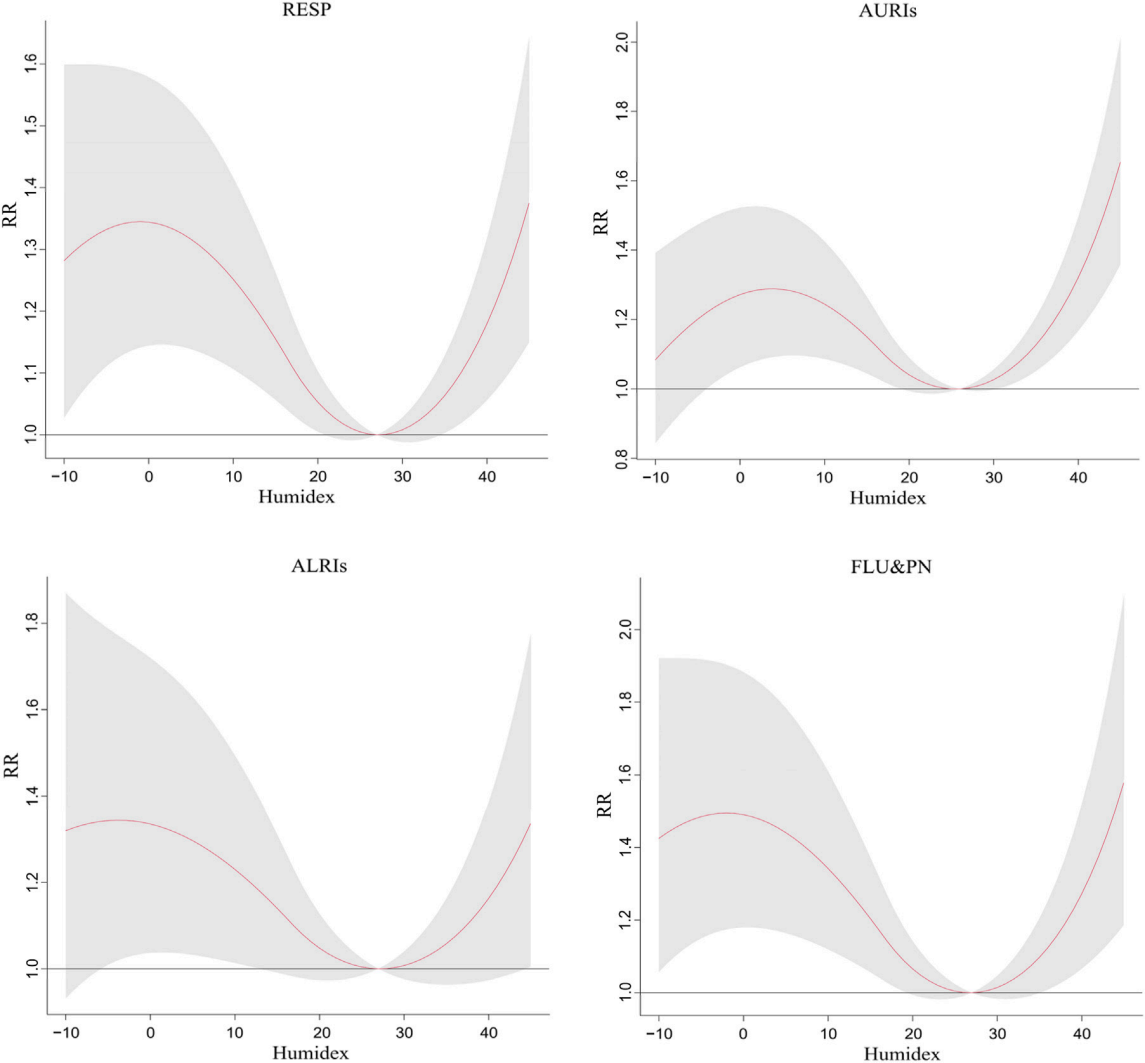
The exposure-response relationship between humidex change and children’s respiratory diseases and their subtypes (Shijiazhuang, China, 2014–2022). Notes: RESP, respiratory diseases; AURIs, acute upper respiratory infections; ALRIs, acute lower respiratory infections; FLU&PN, influenza and pneumonia.

Figure 1 depicted the exposure-response relationship between humidex and the outpatient department of children’s respiratory disorders in Shijiazhuang, with humidex and the outpatient department forming an approximate “V” shape. Both high and low humidex had a significant influence when compared to MRH.

Supplementary Figure S2 displayed the exposure lag response of outpatient visits for respiratory diseases in children with extreme humidex on different lag days. The lagged impact of extremely high humidex on children’s respiratory disease clinics appeared on the first day (RR = 1.012, 95% CI = 1.002–1.022) after exposure and continued until the 8th day (RR = 1.007, 95% CI = 1.000–1.013). However, the lag effect of extremely low humidex occurred from lag 8 to lag 20, with a peak at a lag of about 15 (RR = 1.046, 95% CI = 1.033–1.058). The lag diagram of the impact of extremely high and low humidex on children’s acute upper respiratory infections, acute lower respiratory infections, and influenza and pneumonia outpatient visits in different lag days can be found in Supplementary Figure S2.

Table 4 showed the cumulative relative risk (CRR) of the effects of different extreme humidex on children’s respiratory diseases. We found that both extremely low and high humidex were associated with an increased risk of respiratory diseases in children, with CRR values of 1.344 (95% CI = 1.136–1.590) and 1.124 (95% CI = 1.030–1.228), respectively. There was no significant difference between gender groups. When the humidex was very low, the risk of children aged 7–14 years was significant, with a CRR of 2.148 (95%CI = 1.523–3.030); both extremely low and high humidex were associated with an increased risk of children aged 0–6 years, but they were less affected by extremely low humidex than children aged 7–14 years. In addition, ALRIs were only sensitive to extremely low humidex, with a CRR of 1.342 (95%CI = 1.031–1.748).

**TABLE 4:** The cumulative relative risk of the impact of extreme humidex on outpatient visits of children in different subgroups with lag of 0–21 days (Shijiazhuang, China, 2014–2022).

Variables	MRH	Extremely low humidex	Z	P	Extremely high humidex	Z	P
RESP	27	1.344 (1.136–1.590)			1.124 (1.030–1.228)		
Child’s gender			−0.256	0.399		−0.375	0.354
Boy	27	1.327 (1.118–1.575)			1.113 (1.018–1.217)		
Girl	27	1.371 (1.146–1.640)			1.141 (1.038–1.253)		
Age (in years)			−2.206	0.014		2.996	0.001
0–6	26	1.269 (1.080–1.491)			1.167 (1.065–1.279)		
7–14	37	2.148 (1.523–3.030)			1.001 (0.983–1.02)		
Disease types							
AURIs	26	1.250 (1.035–1.508)	Ref		1.229 (1.112–1.358)	Ref	
FLU&PN	27	1.495 (1.175–1.903)	1.106	0.134	1.188 (1.033–1.367)	−0.387	0.349
ALRIs	27	1.342 (1.031–1.748)	0.420	0.337	1.113 (0.967–1.280)	−1.142	0.127

Notes: RESP, respiratory diseases; AURIs, acute upper respiratory infections; ALRIs, acute lower respiratory infections; FLU&PN, influenza and pneumonia.

Table 5 showed the attributable fractions related to humidex for different subgroups. A total of 13.96% of RESP outpatients were attributed to humidex. Most of the outpatient burden of RESP could be attributed to low humidex, and the corresponding AF was 12.54% (95% eCI = 5.94–18.32%). The composition of attribution burden in different subgroups was consistent with RESP, and the burden of treatment attributed to low humidex was greater.

**TABLE 5:** Attribution fractions of the impact of the humidex on children’s respiratory diseases (Shijiazhuang, China, 2014–2022).

Variables	Humidex	Low humidex	High humidex
RESP	13.96 (7.81–19.33)	12.54 (5.94–18.32)	1.42 (0.19–2.48)
Child’s gender			
Boy	13.48 (6.99–18.91)	12.18 (5.71–17.98)	1.30 (0.04–2.49)
Girl	14.71 (7.67–20.31)	13.11 (5.97–19.19)	1.60 (0.29–2.75)
Age (in years)			
0–6	12.07 (6.14–17.30)	10.12 (3.56–15.78)	1.95 (0.68–3.12)
7–14	30.18 (17.57–39.98)	30.11 (17.45–40.21)	0.07 (−0.26–0.35)
Disease types			
AURIs	13.30 (6.66–18.74)	10.37 (3.72–16.27)	2.93 (1.35–4.30)
FLU&PN	20.66 (10.53–28.95)	19.09 (8.51–27.93)	1.57 (0.18–2.82)
ALRIs	13.75 (2.75–22.28)	12.54 (1.50–21.55)	1.21 (−0.60–2.87)

Notes: RESP, respiratory diseases; AURIs, acute upper respiratory infections; ALRIs, acute lower respiratory infections; FLU&PN, influenza and pneumonia.

## Discussion

Our findings showed that humidex, particularly low humidex, increased the outpatient rate of children’s respiratory diseases in Shijiazhuang. A study in Fuzhou identified a substantial relationship between low temperature, low humidity, and children’s respiratory disease clinics, which is consistent with our findings [11]. In subgroup analysis, our findings revealed that children aged 7–14 were more susceptible to low humidex.

Humidex considered temperature and humidity, which could better understand the human body’s response to climate. Previous research has demonstrated that humidity has a moderating impact on temperature [28]. Furthermore, research has looked into the association between humidex and disease outcomes. For example, a study based on numerous cities in Southwest China found that humidex also significantly increased cardiovascular disease mortality [29]. Another time series study conducted in Hefei was similar to our findings, revealing that low humidex was related to an increased incidence of pediatric asthma [14]. Children are still growing and developing, so their immune systems and physiological organs are not fully formed. Therefore, they are vulnerable to external environmental factors [14, 21]. In addition, low temperature and humidity are conducive to the survival of viruses such as respiratory syncytial virus, thereby increasing the risk of respiratory diseases in children [30]. So far, the probable pathogenic mechanism underlying humidex’s impact on children’s respiratory health remains unknown. Thus, more studies are required to offer biological support.

Our study found that in the first few days of lag, the extremely low humidex had a protective effect on outpatient visits for respiratory diseases in children, which was not consistent with most previous studies [14, 31–33]. However, a time-series study conducted in a northern Chinese city (Harbin) found that extremely low temperature had a protective effect on daily outpatient visits for children with respiratory diseases [10]. The humidex is highly correlated with the daily average temperature. A possible explanation for this result may be that both our study area and Harbin belong to cities in northern China. In order to avoid cold weather, heating equipment has been installed in the homes of urban residents in northern cities, which may lead to protection [10]. Moreover, when the extremely low humidex appears, the reduction of children’s outdoor activities may also lead to inconsistent results. It is noteworthy that with the increase in lag time, the protective effect of extremely low humidex on children’s respiratory disease clinics gradually disappeared, while the lag effect gradually increased with the lag time and lasted for about 2 weeks. Therefore, the lag effect of extremely low humidex should be focused on in the subsequent public health intervention stage.

Age was a crucial element. According to the age stratification analysis, we found that school-age children (7–14 years old) were more susceptible to low humidex. Similar to our results, a study in Hefei found that school-age children were more sensitive to low humidex and more likely to develop asthma than other children [14]. Due to the high activity of school-age children, they may spend more time on outdoor activities, making them more exposed to the outdoor environment for a longer time, resulting in their higher susceptibility to low humidex than preschool children [21]. Studies have confirmed that human immunity will be affected in exposure and low temperature environments, which may increase the risk of respiratory diseases in children [34]. In addition, humidity also plays an important role. When the humidity is low, it will be conducive to the spread of pathogens, such as airway bacteria in the air, coupled with the dryness of the airway mucosa, eventually leading to an increased risk of respiratory diseases [35, 36].

AF is an indicator that quantitatively estimates the effect of exposure factors (temperature, etc.) on population health outcomes (morbidity, hospitalization, or death). It is the proportion of diseases attributable to exposure factors in the population, that is, the proportion of diseases reduced by eliminating risk factors in the population. This indicator is more helpful in estimating the potential benefits of public health interventions and preventive measures. In this study, the AF of children’s respiratory diseases was 13.96%, and the attribution burden caused by low humidex was greater than that caused by high humidex, respectively 12.54% and 1.42%. This result is similar to previous studies [14, 29], indicating that cold weather is more likely to increase the risk of respiratory diseases in children than hot weather. In the subgroup analysis, most AF was attributed to low humidex. This may be related to the higher MRH, which makes the low humidex contain a wider range, resulting in a higher AF. In future research, we can further divide the humidex into different ranges and study its impact on health. In addition, the low humidex covers the winter and spring, and the seasonal alternating temperature changes greatly. Studies have confirmed that changes in temperature can easily induce the incidence and death of weather-sensitive diseases such as the respiratory system [37]. Our findings can provide epidemiological evidence for the development of guidelines for the prevention and treatment of weather-sensitive diseases.

We did not find any difference in the ability of humidex and average temperature to indicate children’s respiratory diseases, and both exposures thus appear to be equally able to predict incidence (Supplementary Table S2).

This study has a few drawbacks. First, similar to other studies, we used data from outdoor fixed environmental monitoring sites for analysis, which may differ from children’s actual exposure level. Second, our research only used data from Shijiazhuang, China. Shijiazhuang belongs to a temperate continental monsoon climate, and the dry and wet periods are apparent. Therefore, caution should be taken when projecting these findings to other regions, particularly cities in varying climate zones.

In conclusion, our findings suggest that humidex exposure raises the incidence of respiratory disease in children. Exposure to low humidex had a greater impact on school-age children. Our findings may spark fresh ideas for future exploration into the relationship between children’s respiratory disorders and the environment.

### Conclusion

This study examined the relationship between humidex and the outpatient visit rate of children’s respiratory diseases in Shijiazhuang, China. Our findings showed that exposure to humidex increases the risk of children’s respiratory diseases, and most of the disease burden can be attributed to low humidex. This study provides a necessary reference for a more comprehensive and quantitative study of respiratory diseases and temperature and humidity indicators in children. Children aged 7–14 years were more sensitive to low humidex. These children need more attention and intervention. When the humidex is low, we can remind people, especially sensitive people, to reduce outdoor activities, pay attention to keeping warm and moisturizing, and use air conditioners, humidifiers, dehumidifiers, and other equipment scientifically.

## References

[1] United Nations. Climate Action: What Is Climate Change? Available online at: https://www.un.org/zh/climatechange/what-is-climate-change (Accessed November 17, 2024).

[2] Li W Luo W. Progress in the Prevention and Treatment of Chronic Respiratory Diseases. Med J West China (2020) 32(01):1–4. 10.3969/i.issn.1672-3511.2020.01.001

[3] GBD 2019 Chronic Respiratory Diseases Collaborators. Global Burden of Chronic Respiratory Diseases and Risk Factors, 1990-2019: An Update from the Global Burden of Disease Study 2019. EClinicalMedicine (2023) 59:101936. 10.1016/j.eclinm.2023.101936 37229504 PMC7614570

[4] Kline O Prunicki M. Climate Change Impacts on Children's Respiratory Health. Curr Opin Pediatr (2023) 35(3):350–5. 10.1097/MOP.0000000000001253 37057656

[5] Feng F Ma Y Zhang Y Shen J Wang H Cheng B Effects of Extreme Temperature on Respiratory Diseases in Lanzhou, a Temperate Climate City of China. Environ Sci Pollut Res Int (2021) 28(35):49278–88. 10.1007/s11356-021-14169-x 33932207

[6] Guo Y Gasparrini A Armstrong BG Tawatsupa B Tobias A Lavigne E Temperature Variability and Mortality: A Multi-Country Study. Environ Health Perspect (2016) 124(10):1554–9. 10.1289/EHP149 27258598 PMC5047764

[7] Chen R Yin P Wang L Liu C Niu Y Wang W Association between Ambient Temperature and Mortality Risk and Burden: Time Series Study in 272 Main Chinese Cities. BMJ (2018) 363:k4306. 10.1136/bmj.k4306 30381293 PMC6207921

[8] Kalkstein LS Greene JS. An Evaluation of Climate/mortality Relationships in Large U.S. Cities and the Possible Impacts of a Climate Change. Environ Health Perspect (1997) 105(1):84–93. 10.1289/ehp.9710584 9074886 PMC1469832

[9] Strosnider HM Chang HH Darrow LA Liu Y Vaidyanathan A Strickland MJ. Age-Specific Associations of Ozone and Fine Particulate Matter with Respiratory Emergency Department Visits in the United States. Am J Respir Crit Care Med (2019) 199(7):882–90. 10.1164/rccm.201806-1147OC 30277796

[10] Wu Y Liu X Gao L Sun X Hong Q Wang Q Short-Term Exposure to Extreme Temperature and Outpatient Visits for Respiratory Diseases Among Children in the Northern City of China: A Time-Series Study. BMC Public Health (2024) 24(1):341. 10.1186/s12889-024-17814-5 38302889 PMC10832290

[11] Wu Z Miao C Li H Wu S Gao H Liu W The Lag-Effects of Meteorological Factors and Air Pollutants on Child Respiratory Diseases in Fuzhou, China. J Glob Health (2022) 12:11010. 10.7189/jogh.12.11010 35973040 PMC9380967

[12] Fang J Song J Wu R Xie Y Xu X Zeng Y Association between Ambient Temperature and Childhood Respiratory Hospital Visits in Beijing, China: A Time-Series Study (2013-2017). Environ Sci Pollut Res Int (2021) 28(23):29445–54. 10.1007/s11356-021-12817-w 33555475

[13] Lai H Lee JE Harrington LJ Ahuriri-Driscoll A Newport C Bolton A Daily Temperatures and Child Hospital Admissions in Aotearoa New Zealand: Case Time Series Analysis. Int J Environ Res Public Health (2024) 21(9):1236. 10.3390/ijerph21091236 39338120 PMC11432253

[14] Pan R Gao J Wang X Bai L Wei Q Yi W Impacts of Exposure to Humidex on the Risk of Childhood Asthma Hospitalizations in Hefei, China: Effect Modification by Gender and Age. Sci total Environ (2019) 691:296–305. 10.1016/j.scitotenv.2019.07.026 31323575

[15] Fang W Li Z Gao J Meng R He G Hou Z The Joint and Interaction Effect of High Temperature and Humidity on Mortality in China. Environ Int (2023) 171:107669. 10.1016/j.envint.2022.107669 36508749

[16] Yin Y Lai M Zhou S Chen Z Jiang X Wang L Effects and Interaction of Temperature and Relative Humidity on the Trend of Influenza Prevalence: A Multi-Central Study Based on 30 Provinces in Mainland China from 2013 to 2018. Infect Dis Model (2023) 8(3):822–31. 10.1016/j.idm.2023.07.005 37496828 PMC10366480

[17] Wang J Zhang L Lei R Li P Li S. Effects and Interaction of Meteorological Parameters on Influenza Incidence during 2010-2019 in Lanzhou, China. Front Public Health (2022) 10:833710. 10.3389/fpubh.2022.833710 35273941 PMC8902077

[18] Nguyen JL Dockery DW. Daily Indoor-To-Outdoor Temperature and Humidity Relationships: A Sample across Seasons and Diverse Climatic Regions. Int J Biometeorol (2016) 60(2):221–9. 10.1007/s00484-015-1019-5 26054827 PMC4674394

[19] Baldwin JW Benmarhnia T Ebi KL Jay O Lutsko NJ Vanos JK. Humidity's Role in Heat-Related Health Outcomes: A Heated Debate. Environ Health Perspect (2023) 131(5):55001. 10.1289/EHP11807 37255302 PMC10231239

[20] d'Ambrosio Alfano FR Palella BI Riccio G. Thermal Environment Assessment Reliability Using Temperature--Humidity Indices. Ind Health (2011) 49(1):95–106. 10.2486/indhealth.ms1097 20823629

[21] Zhao H Yang Y Feng C Wang W Yang C Yin Y Nonlinear Effects of Humidex on Risk of Outpatient Visit for Allergic Conjunctivitis Among Children and Adolescents in Shanghai, China: A Time Series Analysis. J Glob Health (2023) 13:04132. 10.7189/jogh.13.04132 37921044 PMC10623378

[22] Zhang W Du Z Zhang D Yu S Huang Y Hao Y. Assessing the Impact of Humidex on HFMD in Guangdong Province and its Variability across Social-Economic Status and Age Groups. Sci Rep (2016) 6:18965. 10.1038/srep18965 26743684 PMC4705518

[23] Zhang Y Wang SG Ma YX Shang KZ Cheng YF Li X Association between Ambient Air Pollution and Hospital Emergency Admissions for Respiratory and Cardiovascular Diseases in Beijing: A Time Series Study. Biomed Environ Sci (2015) 28(5):352–63. 10.3967/bes2015.049 26055562

[24] Mukaka MM. Statistics Corner: A Guide to Appropriate Use of Correlation Coefficient in Medical Research. Malawi Med J (2012) 24(3):69–71. 23638278 PMC3576830

[25] Akaike H. A New Look at the Statistical Model Identification. IEEE Trans automatic Control (1974) 19(6):716–23. 10.1109/tac.1974.1100705

[26] Gasparrini A Leone M. Attributable Risk from Distributed Lag Models. BMC Med Res Methodol (2014) 14:55. 10.1186/1471-2288-14-55 24758509 PMC4021419

[27] Greenland S. Interval Estimation by Simulation as an Alternative to and Extension of Confidence Intervals. Int J Epidemiol (2004) 33(6):1389–97. 10.1093/ije/dyh276 15319402

[28] Mostofsky E Wilker EH Schwartz J Zanobetti A Gold DR Wellenius GA Short-Term Changes in Ambient Temperature and Risk of Ischemic Stroke. Cerebrovasc Dis extra (2014) 4(1):9–18. 10.1159/000357352 24575110 PMC3934677

[29] Li Y Xia Y Zhu H Shi C Jiang X Ruan S Impacts of Exposure to Humidex on Cardiovascular Mortality: A Multi-City Study in Southwest China. BMC Public Health (2023) 23(1):1916. 10.1186/s12889-023-16818-x 37794404 PMC10548730

[30] Khor CS Sam IC Hooi PS Quek KF Chan YF. Epidemiology and Seasonality of Respiratory Viral Infections in Hospitalized Children in Kuala Lumpur, Malaysia: A Retrospective Study of 27 Years. BMC Pediatr (2012) 12:32–9. 10.1186/1471-2431-12-32 22429933 PMC3337250

[31] Li X Zhang Y Tian Z Wang J Zhao J Lyu Y Lag Effect of Ambient Temperature on Respiratory Emergency Department Visits in Beijing: A Time Series and Pooled Analysis. BMC Public Health (2024) 24(1):1363. 10.1186/s12889-024-18839-6 38773497 PMC11106889

[32] Ma Y Wang H Cheng B Shen J Li H Guo Y Health Risk of Extreme Low Temperature on Respiratory Diseases in Western China. Environ Sci Pollut Res Int (2022) 29(24):35760–7. 10.1007/s11356-021-18194-8 35060041

[33] Chai G He H Su Y Sha Y Zong S. Lag Effect of Air Temperature on the Incidence of Respiratory Diseases in Lanzhou, China. Int J Biometeorol (2020) 64(1):83–93. 10.1007/s00484-019-01795-x 31612311

[34] Schaller M Hogaboam CM Lukacs N Kunkel SL. Respiratory Viral Infections Drive Chemokine Expression and Exacerbate the Asthmatic Response. J Allergy Clin Immunol (2006) 118(2):295–302. 10.1016/j.jaci.2006.05.025 16890750 PMC7172995

[35] Chan PK Mok HY Lee TC Chu IM Lam WY Sung JJ. Seasonal Influenza Activity in Hong Kong and its Association with Meteorological Variations. J Med Virol (2009) 81(10):1797–806. 10.1002/jmv.21551 19697414

[36] Tang JW Lai FY Wong F Hon KL. Incidence of Common Respiratory Viral Infections Related to Climate Factors in Hospitalized Children in Hong Kong. Epidemiol Infect (2010) 138(2):226–35. 10.1017/S0950268809990410 19631018

[37] Bunker A Wildenhain J Vandenbergh A Henschke N Rocklöv J Hajat S Effects of Air Temperature on Climate-Sensitive Mortality and Morbidity Outcomes in the Elderly; a Systematic Review and Meta-Analysis of Epidemiological Evidence. EBioMedicine (2016) 6:258–68. 10.1016/j.ebiom.2016.02.034 27211569 PMC4856745

