# Supplementary material for: The Association Between Humidex and Daily Outpatient Visits for Pediatric Respiratory Diseases in Shijiazhuang, China: A Time Series Analysis
Source: Int J Public Health. 2025 Mar 17;70:1607752. doi: 10.3389/ijph.2025.1607752 (PMC11955390; doi:10.3389/ijph.2025.1607752)
Supplement: Supplementary file 1 [file DataSheet1.docx]

**The association between humidex and daily outpatient visits for pediatric respiratory diseases in Shijiazhuang, China: a time series analysis**

**Table of contents**

**Appendix 1.** formula S1

**Appendix 2.** sensitivity analysis results

**Table S1.** Sensitivity analysis of the effect of humidex on outpatient visits for respiratory diseases in children (Shijiazhuang, China, 2014-2022).

**Table S2.** Humidex compared with daily average temperature (Shijiazhuang, China, 2014-2022).

**Figure S1.** Time series plot for daily pediatric respiratory outpatient visits, humidex, and meteorological factors (Shijiazhuang, China, 2014-2022).

**Figure S2.** The relationship between extreme humidex and outpatient visits of respiratory diseases in children, categorised by type of respiratory diseases, over various lag days (Shijiazhuang, China, 2014-2022).

**Appendix 1.** formula S1

The formula as follows:

$$Z=\frac{\beta_{1}-\beta_{2}}{\sqrt{SE_{1}^{2}+SE_{2}^{2}}}$$

Where *β*_1_, *β*_2_ are the estimates for two categories (such as boy and girl), and *SE*_1_, *SE*_2_ represent the corresponding standard errors.

**Appendix 2.** sensitivity analysis results

In this sensitivity assessment, we changed the parameter settings of time (df=8-9) and wind (df=3-5), it was found that the attribution risk did not change much, indicating that the model had good stability (Table S1).

**Table S1.** Sensitivity analysis of the effect of humidex on outpatient visits for respiratory diseases in children (Shijiazhuang, China, 2014-2022).

| Degree of freedom | AF（95%*CI*） |
| --- | --- |
| time=8、wind=3 | 13.96（7.81 - 19.33） |
| time=8、wind=4 | 13.95（7.43 - 19.23） |
| time=8、wind=5 | 14.00（7.21 - 19.33） |
| time=9、wind=3 | 12.81（-4.18 - 26.81） |
| time=9、wind=4 | 12.92（-4.99 - 26.98） |
| time=9、wind=5 | 12.49（-4.66 - 26.15） |

Notes: AF—attributable fraction.

**Table S2.** Humidex compared with daily average temperature (Shijiazhuang, China, 2014-2022).

| Variables | Humidex | Average temperature | Z | P |
| --- | --- | --- | --- | --- |
| 5th | **1.344（1.136 - 1.590）** | 1.213（0.983 - 1.497） | 0.749 | 0.227 |
| 95th | **1.124（1.030 - 1.228）** | 1.064（0.963 - 1.175） | 0.811 | 0.209 |
| AF | **13.96（7.81 - 19.33）** | 11.89（-2.90 - 25.11） | 0.268 | 0.394 |
| AN | **283,299** | 241,291 | — | — |

Notes: 5th—extremely low humidex (-2) and average temperature (-2℃); 95th—extremely high humidex (38) and average temperature (29℃); AF—attributable fraction; AN—attributable number.

**Figure S1.** Time series plot for daily pediatric respiratory outpatient visits, humidex, and meteorological factors (Shijiazhuang, China, 2014-2022). Notes: resp—respiratory diseases.
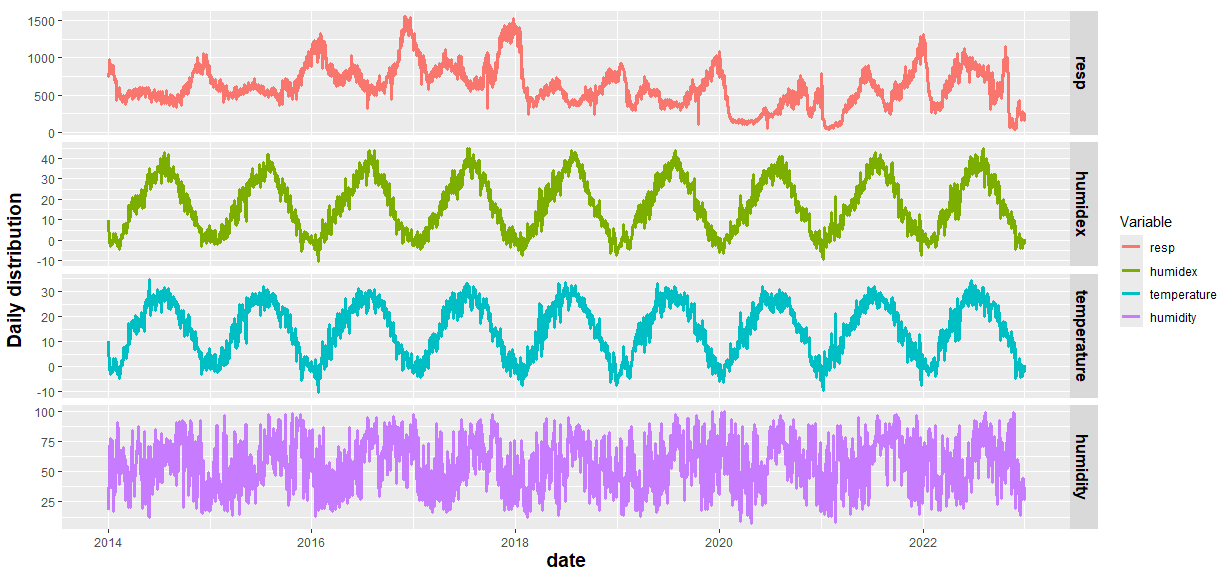


|  | 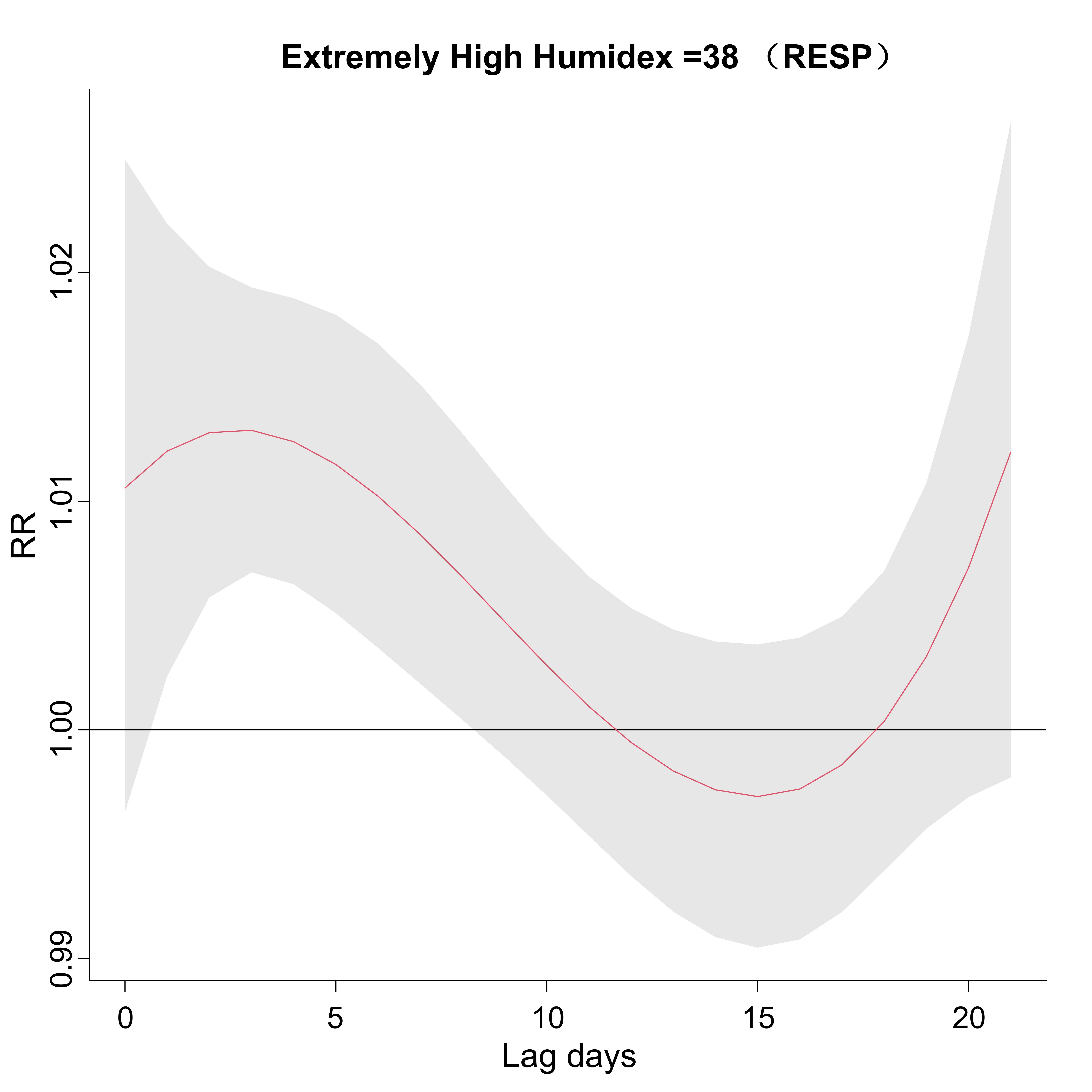 | 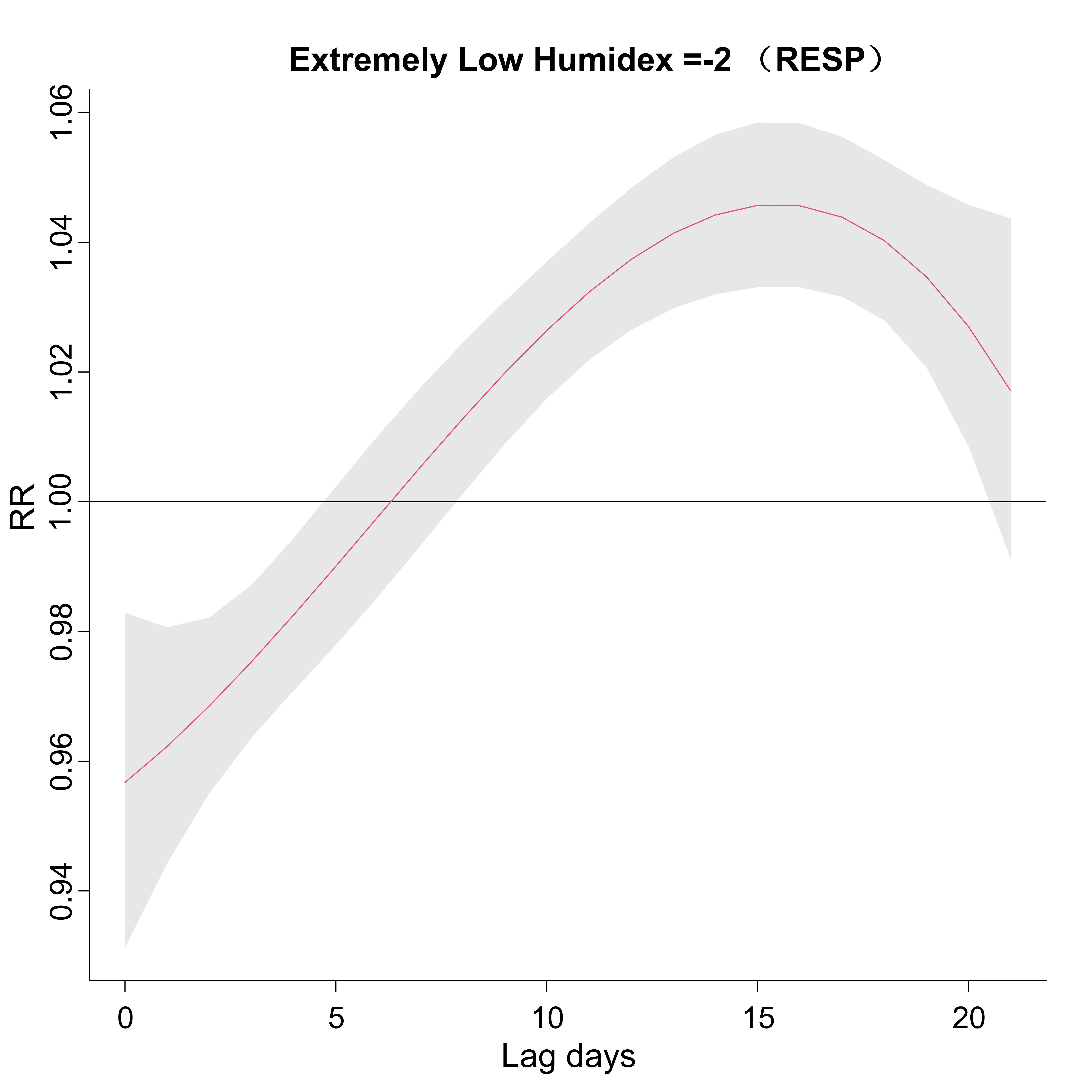 |
| --- | --- | --- |
|  | 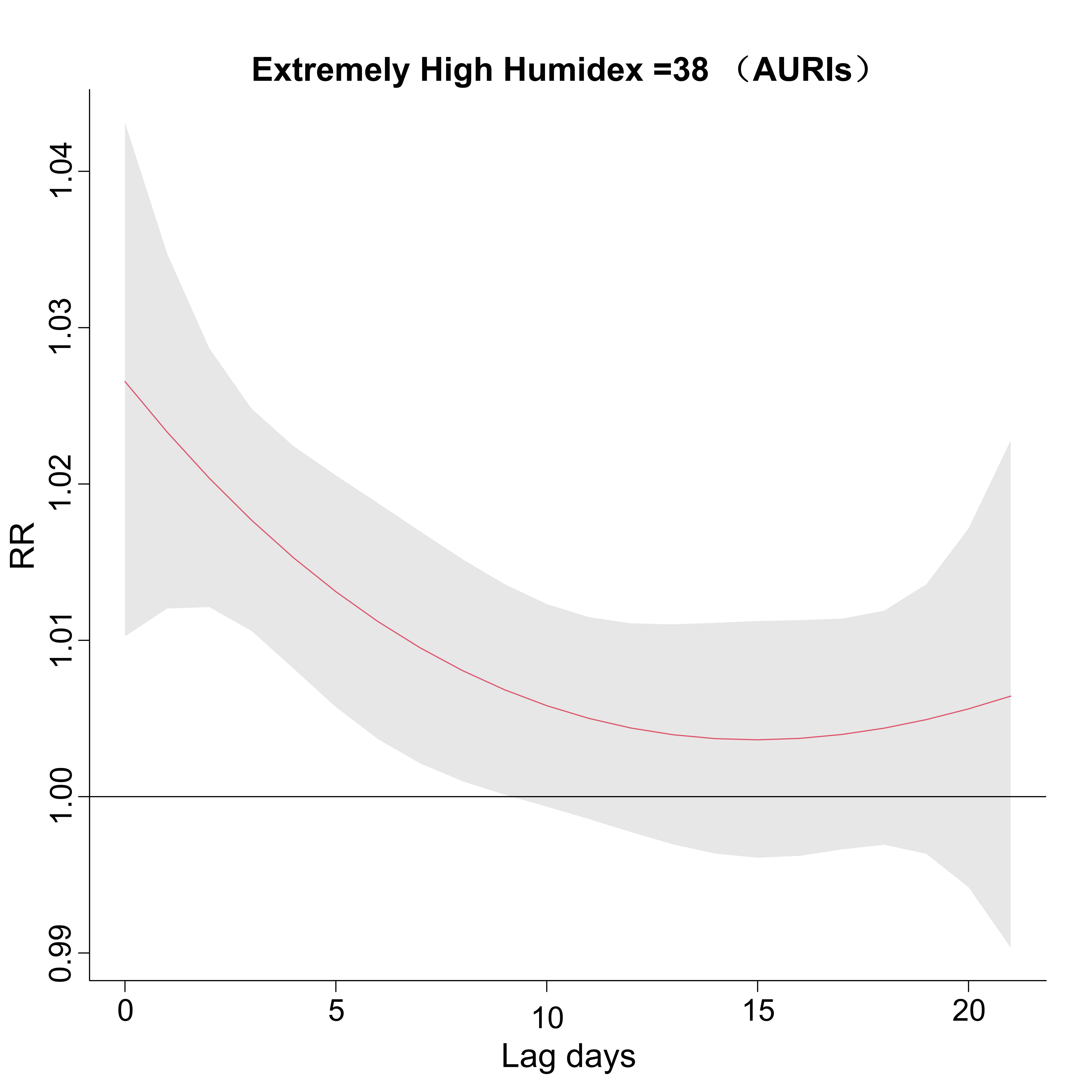 | 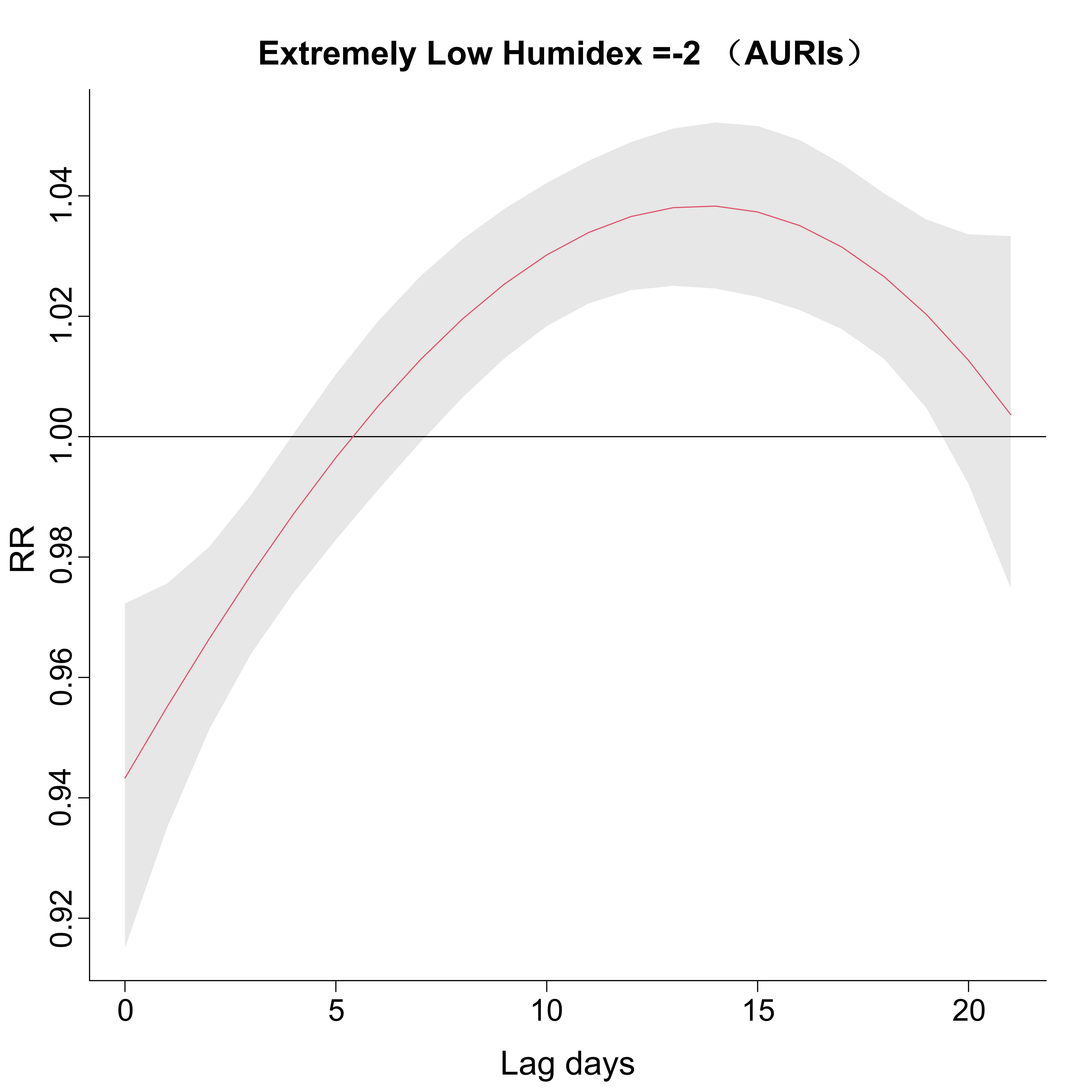 |
|  | 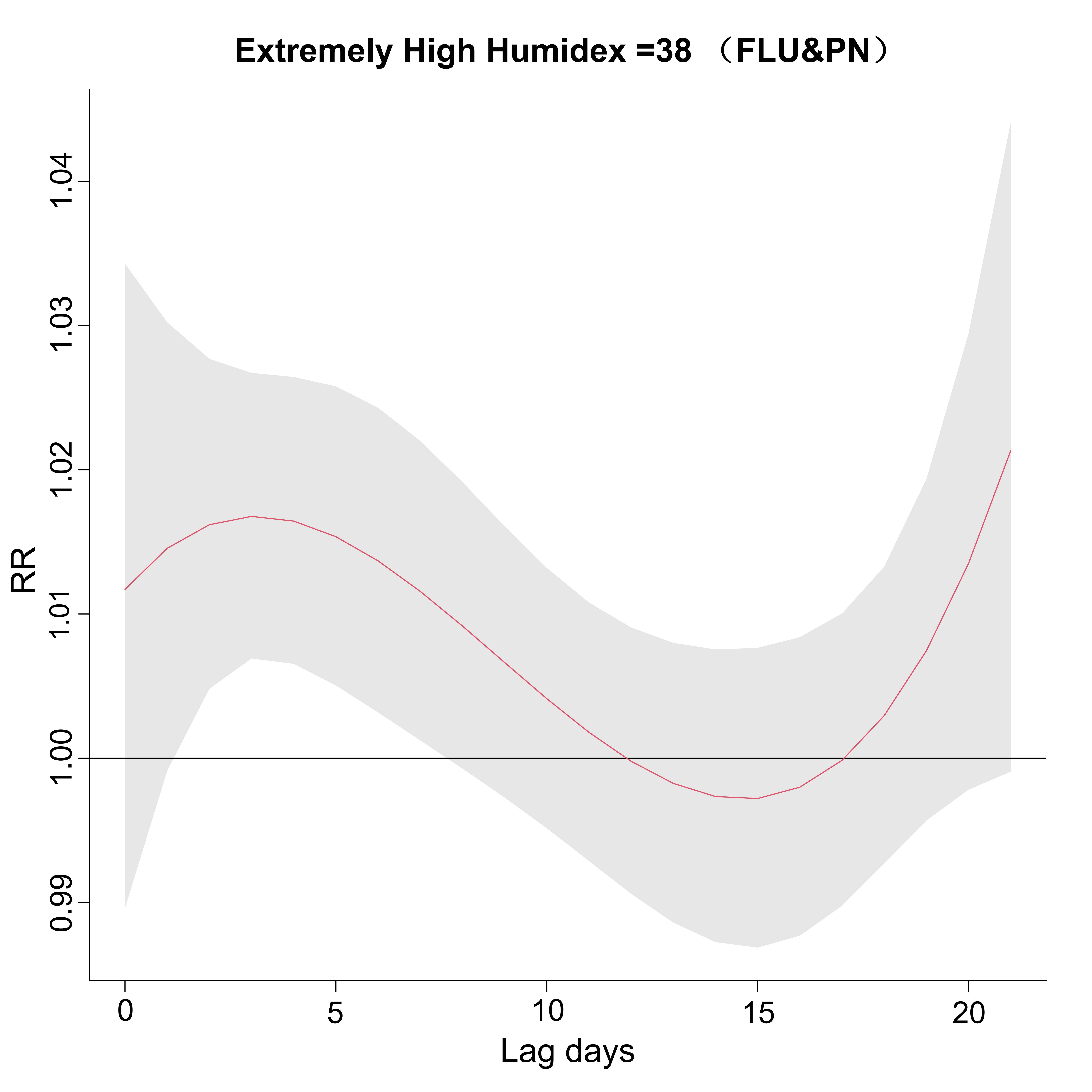 | 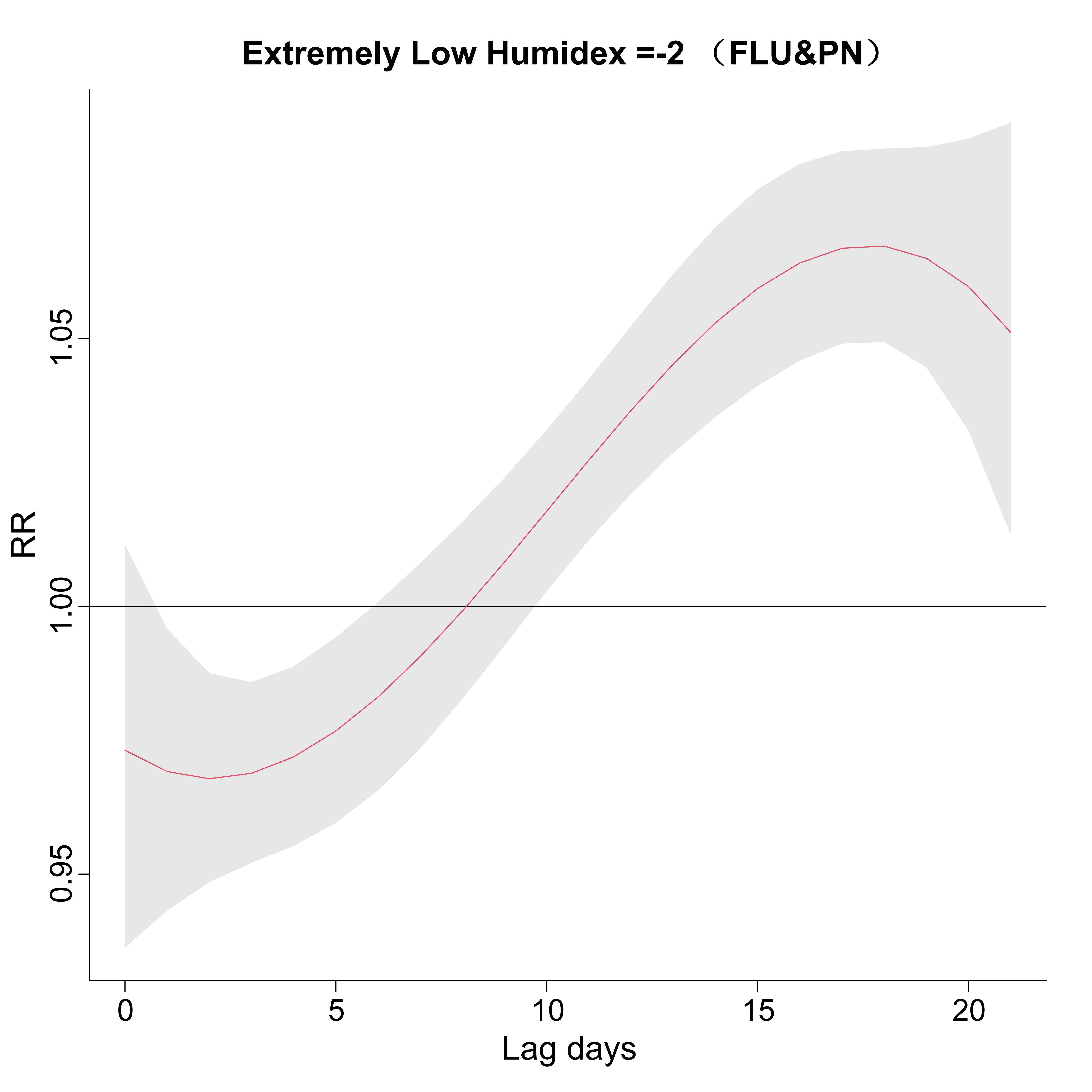 |
|  | 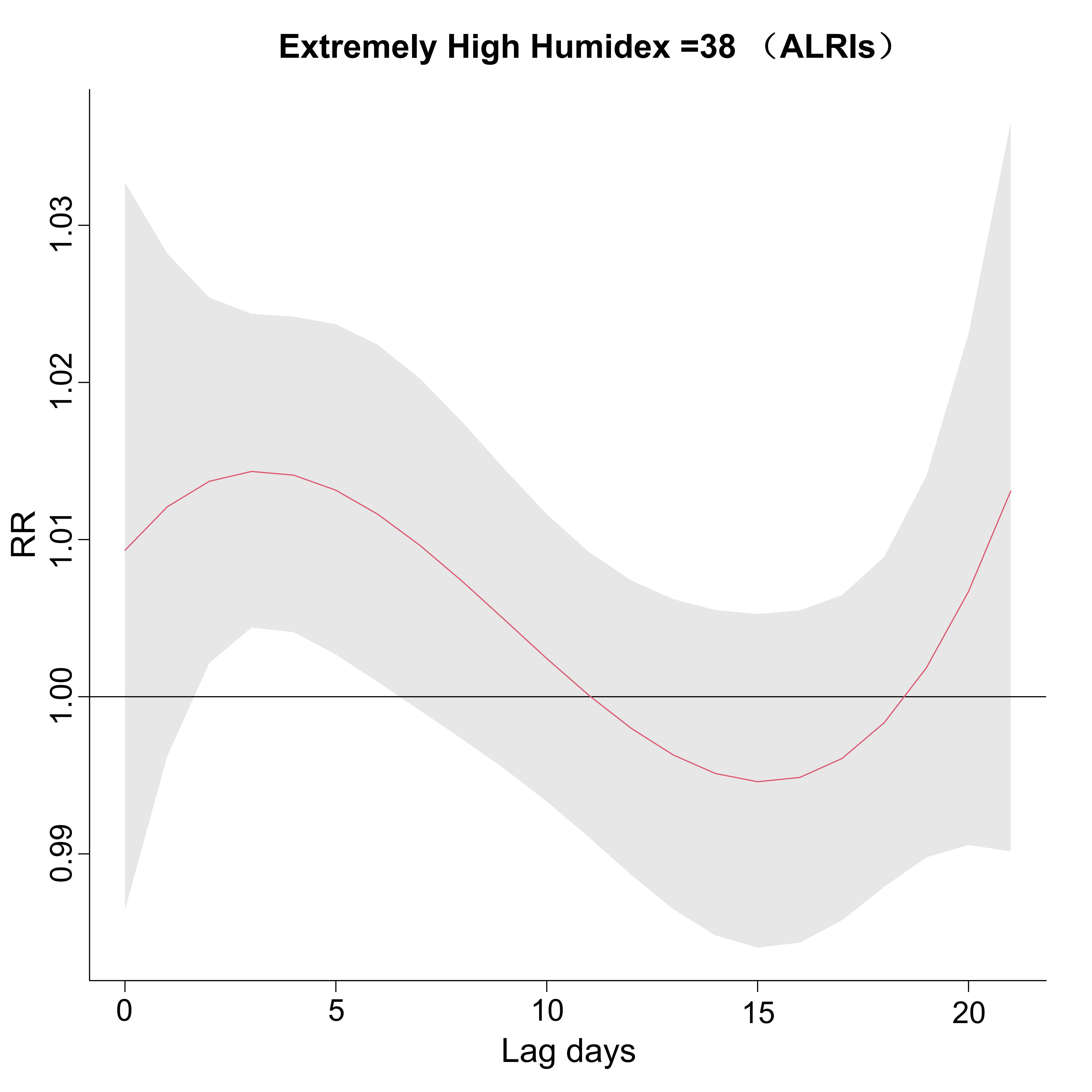 | 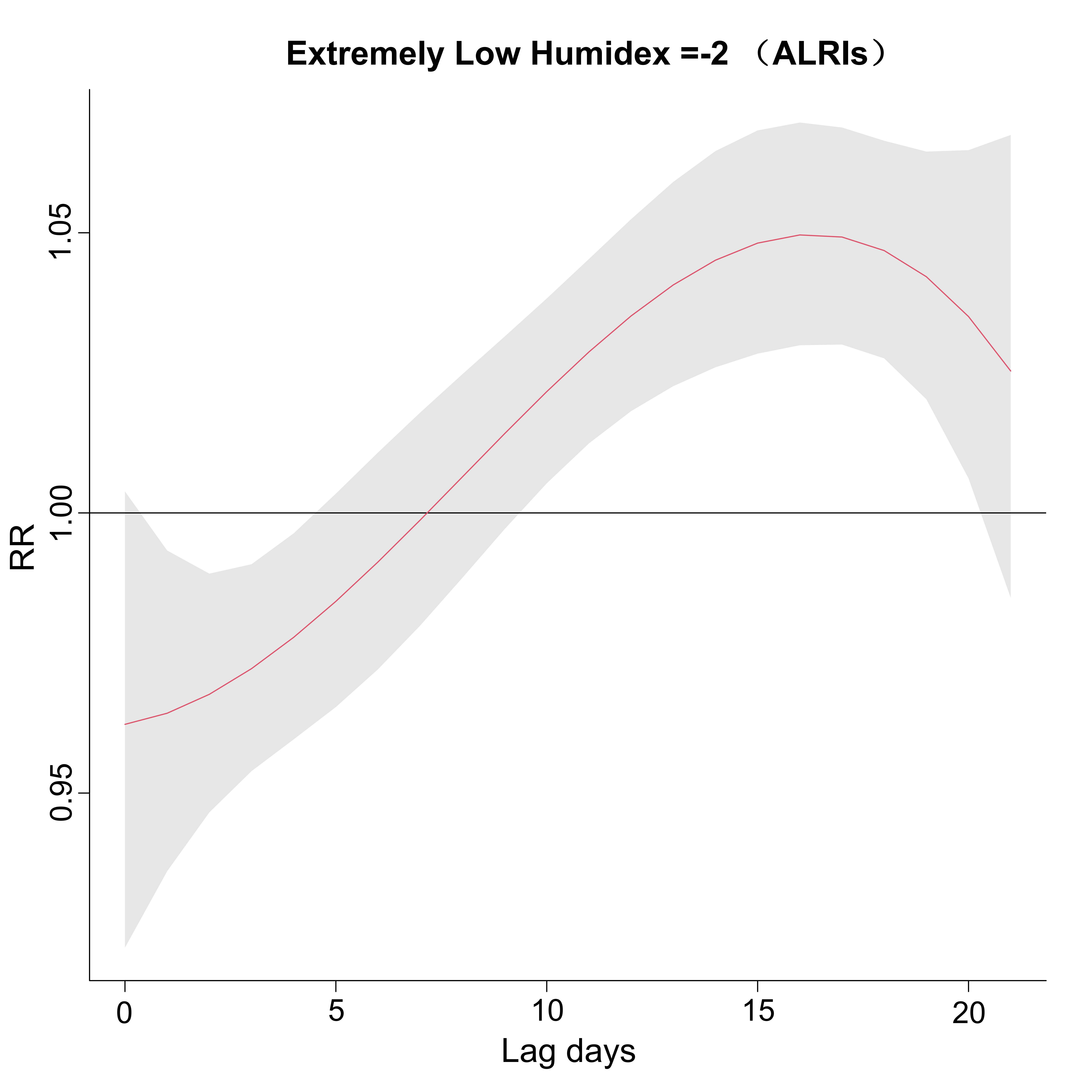 |

**Figure S2.** The relationship between extreme humidex and outpatient visits of respiratory diseases in children, categorised by type of respiratory diseases, over various lag days (Shijiazhuang, China, 2014-2022). Notes: RESP—respiratory diseases; AURIs—acute upper respiratory infections; ALRIs—acute lower respiratory infections; FLU&PN—influenza and pneumonia.
